# Efficacy of a meal replacement diet plan compared to a food-based diet plan after a period of weight loss and weight maintenance: a randomized controlled trial

**DOI:** 10.1186/1475-2891-9-11

**Published:** 2010-03-11

**Authors:** Lisa M Davis, Christopher Coleman, Jessica Kiel, Joni Rampolla, Tammy Hutchisen, Laura Ford, Wayne S Andersen, Andrea Hanlon-Mitola

**Affiliations:** 1 Research & Development, Medifast, Inc, Owings Mills, Maryland, USA; 2 Private Practice, Clifton Park, New York, USA

## Abstract

**Background:**

Obesity has reached epidemic proportions in the United States. It is implicated in the development of a variety of chronic disease states and is associated with increased levels of inflammation and oxidative stress. The objective of this study is to examine the effect of Medifast's meal replacement program (MD) on body weight, body composition, and biomarkers of inflammation and oxidative stress among obese individuals following a period of weight loss and weight maintenance compared to a an isocaloric, food-based diet (FB).

**Methods:**

This 40-week randomized, controlled clinical trial included 90 obese adults with a body mass index (BMI) between 30 and 50 kg/m^2^, randomly assigned to one of two weight loss programs for 16 weeks and then followed for a 24-week period of weight maintenance. The dietary interventions consisted of Medifast's meal replacement program for weight loss and weight maintenance, or a self-selected, isocaloric, food-based meal plan.

**Results:**

Weight loss at 16 weeks was significantly better in the Medifast group (MD) versus the food-based group (FB) (12.3% vs. 6.9%), and while significantly more weight was regained during weight maintenance on MD versus FB, overall greater weight loss was achieved on MD versus FB. Significantly more of the MD participants lost ≥ 5% of their initial weight at week 16 (93% vs. 55%) and week 40 (62% vs. 30%). There was no difference in satiety observed between the two groups during the weight loss phase. Significant improvements in body composition were also observed in MD participants compared to FB at week 16 and week 40. At week 40, both groups experienced improvements in biochemical outcomes and other clinical indicators.

**Conclusions:**

Our data suggest that the meal replacement diet plan evaluated was an effective strategy for producing robust initial weight loss and for achieving improvements in a number of health-related parameters during weight maintenance, including inflammation and oxidative stress, two key factors more recently shown to underlie our most common chronic diseases.

**Trial Registration:**

ClinicalTrials.gov NCT01011491

## Background

Obesity is a chronic, complex, multifactorial disorder [1] that has reached epidemic proportions in the United States. Currently, an estimated 66% of the population is categorized as overweight or obese, and 32.2% obese [2]. Obesity is associated with an increased risk of morbidity and mortality secondary to complicating conditions that include heart disease, diabetes, cancer, asthma, sleep apnea, arthritis, reproductive complications, and psychological disturbances [3]. Moreover, obesity is associated with greater degrees of inflammation and oxidative stress [4], which have recently been shown to underlie many chronic conditions, from cardiovascular disease and cancer [5], to metabolic syndrome and nonalcoholic fatty liver disease [6], to neurodegenerative diseases, like Parkinson's disease [7]. Given the prevalence of obesity, its harmful consequences on human health, and the lack of effective treatment options, meal replacement diet plans represent a viable strategy for controlling weight and positively impacting health outcomes.

Results of our previous research [8] as well as that of others [9] demonstrate the safety and efficacy of meal replacements for weight loss and weight maintenance among overweight and obese individuals. Evidence has shown that dietary interventions utilizing meal replacements result in greater weight loss [9] better compliance [8,10], are more likely to ensure adequate intake of essential nutrients [10,11], and demonstrate higher satisfaction and lower drop-out rates compared to other diets [8,9,11,12].

Previous studies have also found improvements in biochemical markers over both the short-term (3-months) and the long-term (≥ 27 months) [13-15] when meal replacements were used as part of a hypocaloric diet. More recently, meal replacement diet plans have been shown to improve levels of C-reactive protein, a biomarker of systemic inflammation [16,17]. Increased body weight, percent body fat, and waist circumference have been positively correlated with levels of C-reactive protein [18]. Individuals categorized as overweight (BMI: 25-29 kg/m^2^) have been shown to have higher levels of CRP compared to lean individuals BMI (<25 kg/m^2^) [19]. Elevated levels of CRP are associated with an increased risk for insulin resistance, endothelial dysfunction [20], oxidative stress [21], and cardiovascular events [22]. Calorie-restricted weight loss has been shown to decrease CRP concentrations [16,17,22]. The loss of body weight, particularly around the abdomen, may lower the risk of chronic diseases like cardiovascular disease by dampening systemic inflammation [4,5] and reducing levels of oxidative stress [23].

Thus, several lines of evidence suggest that hypocaloric meal replacement diet plans may be an effective strategy for fostering weight loss, ensuring compliance, and improving health outcomes in today's obesigenic environment. We therefore sought to evaluate the impact of a previously untested portion-controlled meal replacement diet plan on body weight and body composition compared to an isocaloric, food-based diet plan during a 16-week period of weight loss and 24-week period of weight maintenance. Given the scarcity of existing research evaluating the impact of meal replacements on inflammation and oxidative stress, these biomarkers were also collected as secondary outcomes.

## Methods

### Data Collection Procedures

All participants gave written informed consent, and the protocol was approved by the Western Institutional Review Board.

Participants were obese (BMI > 30.0 kg/m^2 ^≤ 50.0 kg/m^2^) men and women aged 18-65 who were interested in weight loss, but not actively involved in a weight loss program or losing weight. Some Medifast meal replacements contain soy, wheat, gluten and nuts so we ensured participants had no known allergies to these ingredients. To avoid the potential affects on calorie intake and compliance, participants consumed ≤ 14 alcoholic beverages per week and agreed to avoid alcohol intake during the study. Participants were not currently using appetite-affecting medications [e.g selective serotonin reuptake inhibitors (SSRIs), steroids, Ritalin], and were not pregnant or lactating. Participants were required to have a normal electrocardiogram (EKG) and lab work within the past year as well as the permission of their primary care provider to enroll in the study. Subjects were recruited using flyers, newspaper advertisements, and Craigslist.

Exclusion criteria included individuals that were actively dieting; had chronic uncontrolled health problems (not including obesity or diabetes); had a pacemaker or other internal electronic medical device; reported schizophrenia, history of bipolar disorder, or a current Major Depressive Disorder; dependence on alcohol or sedative-hypnotic drugs (e.g. benzodiazepines); cognitive impairment severe enough to preclude informed consent; or who were currently taking weight loss or appetite affecting medications. Major eating disorders were screened using the Eating Attitudes Test (EAT). A score of > 30 was exclusionary.

Individuals meeting initial eligibility criteria by phone were invited for in-person screening at a suburban Baltimore clinical research facility. At this visit, written informed consent and Health Insurance Portability and Accountability Act (HIPAA) authorization were obtained. Measurements of height, weight, waist circumference (WC), blood pressure, pulse, and body composition using bioelectrical impedance (BIA) were collected. The EAT screening tool was administered. Data on general demographics, medical history, weight history, alcohol and cigarette use, exercise, eating habits, and sources of stress were collected.

One hundred fifteen obese adults met initial eligibility criteria and attended the in-person screening. Six individuals withdrew immediately following screening, 2 individuals were excluded during the in-person screening visit, and 17 individuals failed to attend the baseline visit resulting in a final sample of 90 participants (64 women, 26 men).

### Intervention

Participants were randomly assigned to follow one of two hypocaloric (providing less than estimated calorie needs as determined by the Mifflin-St. Jeor equation) weight loss plans for a time period of 16-weeks: the Medifast 5 & 1 Plan (MD) (Medifast, Inc, Owings Mills, MD) utilizing portion-controlled meal replacements or an isocaloric food-based plan (FB) using guidelines from the USDA Food Guide Pyramid, both providing ~1000 kilocalories (kcal) per day. At the baseline visit, a registered dietitian reviewed the dietary intervention each participant was randomized to. Members of the study staff and study participants were not able be blinded to the type of diet, though participants received identical interventions and staff attention. After the initial 16-week weight loss phase both groups entered a 24-week long maintenance phase (for a total of 40 weeks), gradually increasing calorie intake to a maintenance energy level. The MD group continued to utilize meal replacements during the weight maintenance phase.

The intervention diet plan (Medifast 5&1 plan) included 5 meal replacements (90-110 kcal/each), 5-7 oz lean protein, 1 1/2 cups of non-starchy vegetables, and up to 2 fat servings daily (providing 800-1000 kcal). The meal replacements used in this study were low fat, low glycemic index (GI), low sugar, provided a balanced ratio of carbohydrates to proteins, and were either soy and/or whey protein based. The FB plan included 3 ounces of grains, 1 cup of vegetables, 1 cup of fruit, 2 cups of milk, 5-7 ounces of lean protein, and 3 teaspoons of fat daily (providing ~1000 kcal/day). The FB group was also instructed to take a multivitamin and additional calcium to ensure micronutrient needs were met while following a low-calorie meal plan. Vitamin and mineral fortification of the Medifast meals precluded the need for additional supplementation in the MD group.

The Mifflin-St. Jeor equation was used to estimate total daily energy requirements and develop individualized meal plans during the 24-week weight maintenance phase. The FB group followed meal plans based on USDA Food Guide Pyramid guidelines and their estimated energy needs while the MD group was provided a maintenance meal plan incorporating 3-5 meal replacements, depending on the daily energy requirement of the individual. Maintenance phase meal plans were reviewed with each participant by a registered dietitian prior to the beginning of the maintenance phase.

Physical activity above normal daily activities was not a requirement for participation in the study. While following the 5 & 1 plan, 45 minutes of exercise per day above normal daily activities, is the recommended maximum. This same guideline was recommended to the FB group during the weight loss phase. No specific guidelines for physical activity were provided during the weight maintenance phase of the study.

Each participant met with a dietitian bi-weekly during the 16-week weight loss phase for dietary and behavioral counseling and at 12 week intervals during the 24 week weight maintenance phase (weeks 28 and 40 of the study, respectively). Five different dietitians were used to counsel subjects. Each dietitian had subjects from both groups and reviewed identical information with each subject. Every effort was made to have the subjects see the same dietitian throughout the study; however, it was made clear at screening and throughout the study that an alternate dietitian could be requested for any reason until a suitable match was found. At each visit, all participants were provided a self-study module focusing on a behavioral component of weight loss (e.g. stress management).

Participants in the MD group received 40 weeks of meal replacements (16 week supply for weight loss and 24 week supply for weight maintenance) free of cost. After the study was completed, the FB group had the option of receiving an equivalent amount of meal replacements free of cost or a cash payment of $375.

### Measurements

Baseline measures for weight, blood pressure, waist circumference (WC), and body composition [percent body fat, lean muscle mass (LMM) and visceral fat rating (VFR)] were obtained. Bioelectrical impedance (BIA) was used to determine body composition using Tanita's Iron Man BC-549 scale. VFR was determined by an algorithm based on BIA results that generates a rating - the amount of visceral fat itself is not measured. The range for the VFR is 0-59 with a healthy level of visceral fat receiving a rating of 0-12 and an excess level of visceral fat receiving a rating of 13-59. Weight and blood pressure were measured bi-weekly during the 16-week weight loss phase and at 12 week intervals during the maintenance phase (weeks 28 and 40 of the study, respectively). WC, pulse, and body composition were measured at weeks 4, 8, 12, 16, 28 and 40.

A lipid panel, C-reactive protein (CRP), and urine lipid peroxides (ULP) were measured at baseline, at the end of the weight loss phase (week 16) and at the end of weight maintenance (week 40). CRP was used as a biomarker of inflammation and ULP was used as a biomarker of oxidative stress. Lipid panels and CRPs were drawn at a LabCorp patient services center of the participant's choice http://www.labcorp.com. Low levels of CRP were defined as ≤ 3.0 mg/dL and high were defined as > 3.0 mg/dL, which is above the upper limit of normal for LabCorp. Urine samples were collected and sent to Genova Diagnostics http://www.gdx.net for analysis.

Satiety (post-meal fullness and general fullness) was assessed using a 100 mm visual analog scale (VAS) anchored by extremes of fullness. Participants rated their level of fullness over the previous 2 weeks, at baseline and week 16.

### Statistical Analysis

To have a 90 percent chance of detecting a 2% difference between the two diet groups in percentage of initial body weight lost at 16 weeks, with an assumed standard deviation of 5% and a noncompletion rate of 30%, 90 participants were required to be randomized (2 sided, α = .05) to one of the two groups.

Between group differences in demographic, anthropometric and biochemical variables were investigated using χ^2^for categorical variables and non-parametric tests for continuous variables (e.g., Mann-Whitney U). Non-parametric tests were used due to the non-normal distribution of the sample's data for most outcome variables. To examine bivariate longitudinal changes, Wilcoxon signed-rank tests were employed. Random effects logistic regression models were used to examine the association between diet group and outcome variables (i.e., anthropometric and biochemical indices while controlling for confounding variables). Random effects regression allows for a subject-specific interpretation, and adjustment for excess between-individual heterogeneity. Where results did not differ between bivariate t-tests and random effects analyses, only t-test results are shown. Significance was defined as p < 0.05. Analyses were conducted using SPSS Version 15 [24] and Stata Version 10 [25].

## Results

### Subjects

Of the 90 eligible participants (MD = 45, FB = 45) who began the diet, 48 (53%) completed the 16-week active weight loss phase. These included 28 of 45 (62.2%) randomized to the MD group and 20 of 45 (55.6%) randomized to the FB group (χ^2 ^= 2.857, df = 1, p = 0.091). At week 40, after completion of 24 weeks of weight maintenance, 46 participants remained in the study, 26 MD (57.8%) and 20 FB (55.6%) (χ^2 ^= 1.601, df = 1, p = 0.206). MD had significantly higher baseline urine ULPs than FB group (p = 0.05), otherwise there were no significant differences at baseline in other outcome measures. There were no significant adverse events in either group. Baseline characteristics are shown in Table 1. Results are shown (Tables 2, 3, 4, 5) for completers at each stage (post 16-week weight loss, post 24-weeks maintenance phase (week 40)).

**Table 1:** Baseline Characteristics

Demographics		MD Group (n = 45)	FB Group (n = 45)	Between Group p-value
**Age (years)**		43.0 ± 10.2	45.1 ± 11.6	P = 0.362
				
**Gender**	Male	15 (66.7%)	11 (24.4%)	P = 0.352
	Female	30 (33.3%)	34 (75.6%)	
				
**Ethnicity**	Caucasian	27 (60.0%)	19 (42.2%)	P = 0.224
	African Am	16 (35.6%)	25 (55.6%)	
	Hispanic	1 (2.2%)	0 (0.0%)	
	Mixed	1 (2.2%)	1 (2.2%)	

**Table 2:** Anthropometric Measures at Week 0 and Week 16

Measurement	Week	MD Group Mean ± SD	Within Group Δ p-value	Week	FB Group Mean ± SD	Within Group Δ p-value	Between Group Δ p-value
Weight (kgs)*	0 (n = 45)	111.6 ± 25.7	p < 0.0001	0 (n = 45)	104.1 ± 17.1	p < 0.0001	p = 0.001
	16 (n = 28)	98.0 ± 23.9		16 (n = 20)	95.4 ± 18.9		
							
**BMI (kg/m2)**	0 (n = 45)	38.5 ± 6.8	p < 0.0001	0 (n = 45)	37.8 ± 4.5	p = 0.001	p = 0.001
	16 (n = 28)	33.8 ± 6.6		16 (n = 20)	34.7 ± 5.9		
							
**Waist **	0 (n = 45)	116.6 ± 15.5	p < 0.0001	0 (n = 45)	114.2 ± 15.0	p = 0.003	p = 0.028
**Circumference (cm)**	16 (n = 26)	104.5 ± 16.9		16 (n = 20)	104.9 ± 14.9		
							
Body Fat (%)*	0 (n = 45)	42.8 ± 7.7	p < 0.0001	0(n = 45)	44.1 ± 6.4	p = 0.268	p < 0.0001
	16 (n = 27)	37.5 ± 8.8		16 (n = 20)	41.0 ± 7.6		
							
**Lean Muscle **	0 (n = 41)	56.8 ± 11.4	p < 0.0001	0 (n = 41)	53.3 ± 8.7	p = 0.003	p = 0.56
**Mass (kgs)**	16 (n = 25)	55.0 ± 10.4		16 (n = 20)	52.8 ± 9.4		
							
**Visceral Fat **	0 (n = 41)	13.8 ± 3.8	p < 0.0001	0 (n = 41)	13.7 ± 5.0	p = 0.081	p < 0.0001
**Rating**	16 (n = 25)	10.6 ± 3.5		16 (n = 20)	14.2 ± 6.1		
							
**Systolic BP **	0 (n = 45)	125.4 ± 13.9	p < 0.0001	0 (n = 45)	125.8 ± 13.6	p = 0.003	p = 0.667
**(mmHg)**	16 (n = 28)	113.6 ± 14.3		16 (n = 20)	115.8 ± 12.3		
							
**Diastolic BP **	0 (n = 45)	83.2 ± 9.5	p = 0.001	0 (n = 45)	82.6 ± 10.2	p = 0.016	p = 0.622
**(mmHg)**	16 (n = 28)	74.2 ± 8.8		16 (n = 20)	74.2 ± 6.8		
							
**Pulse, **	0 (n = 45)	76.4 ± 9.3	p = 0.001	0 (n = 45)	73.9 ± 9.2	p = 0.011	p = 0.112
**(bpm)**	16 (n = 26)	67.2 ± 8.6		16 (n = 19)	71.2 ± 8.1		

Δ = Change from baseline to 16 weeks.

* = Primary outcomes. All others are secondary outcomes.

**Table 3:** Anthropometric Measures at Week 0 and Week 40

Measurement	Week	MD Group Mean ± SD	Within Group Δ p-value	Week	FB Group Mean ± SD	Within Group Δ p-value	Between Group Δ p-value
**Weight **	0 (n = 45)	111.6 ± 25.7	p < 0.0001	0 (n = 45)	104.1 ± 17.1	p = 0.004	p = 0.18
(kgs)*	40 (n = 26)	103.6 ± 25.2		40 (n = 20)	96.2 ± 19.6		
							
**BMI **	0 (n = 45)	38.5 ± 6.8	p < 0.0001	0 (n = 45)	37.8 ± 4.5	p = 0.003	p = 0.18
**(kg/m2)**	40 (n = 26)	35.6 ± 7.4		40 (n = 20)	35.0 ± 6.2		
							
**Waist **	0 (n = 45)	116.6 ± 15.5	p < 0.0001	0 (n = 45)	114.2 ± 15.0	p = 0.121	p = 0.026
**Circumference (cm)**	40 (n = 25)	106.4 ± 17.3		40 (n = 17)	109.9 ± 15.4		
							
Body Fat (%)*	0 (n = 45)	42.8 ± 7.7	p < 0.0001	0 (n = 45)	44.1 ± 6.4	p = 0.103	p = 0.074
	40 (n = 26)	39.9 ± 8.1		40 (n = 20)	40.7 ± 9.1		
							
**Lean Muscle **	0 (n = 41)	56.8 ± 11.4	p = 0.008	0 (n = 41)	53.3 ± 8.7	p = 0.009	p = 0.795
**Mass (kg)**	40 (n = 24)	55.0 ± 10.0		40 (n = 20)	53.2 ± 10.0		
							
**Visceral Fat **	0 (n = 41)	13.8 ± 3.8	p < 0.0001	0 (n = 41)	13.7 ± 5.0	p = 0.039	p = 0.102
**Rating**	40 (n = 24)	12.0 ± 4.2		40 (n = 20)	13.7 ± 6.0		
							
**Systolic BP **	0 (n = 45)	125.4 ± 13.9	p = 0.009	0 (n = 45)	125.8 ± 13.6	p = 0.016	p = 0.373
**(mmHg)**	40 (n = 26)	117.2 ± 13.7		40 (n = 20)	116.7 ± 14.8		
							
**Diastolic BP (mmHg)**	0 (n = 45)	83.2 ± 9.5	p = 0.009	0 (n = 45)	82.6 ± 10.2	p = 0.618	p = 0.053
**(mmHg)**	40 (n = 26)	75.1 ± 10.6		40 (n = 20)	78.5 ± 9.2		
							
**Pulse **	0 (n = 45)	76.4 ± 9.3	p = 0.004	0 (n = 45)	73.9 ± 9.2	p = 0.025	p = 0.324
**(bpm)**	40 (n = 26)	69.8 ± 8.4		40 (n = 20)	72.4 ± 6.8		

Δ = Change from baseline to 40 weeks.

* = Primary outcomes. All others are secondary outcomes.

**Table 4:** Lipid and Inflammatory Measures at Week 0 and Week 16

Measurement^†^	Week	MD Group Mean ± SD	Within Group Δ p-value	Week	FB Group Mean ± SD	Within Group Δ p-value	Between Group Δ p-value
**Total Cholesterol **	0 (n = 39)	191.2 ± 39.5	p = 0.841	0 (n = 40)	187.5 ± 43.0	p = 0.687	p = 0.409
**(mg/dl)**	16 (n = 25)	181.3 ± 40.4		16 (n = 20)	184.9 ± 45.9		
							
**HDL **	0 (n = 39)	52.0 ± 16.0	p = 0.843	0 (n = 40)	51.8 ± 18.0	p = 0.261	p = 0.62
**(mg/dl)**	16 (n = 25)	51.6 ± 11.3		16 (n = 20)	48.2 ± 11.3		
							
**LDL **	0 (n = 39)	117.3 ± 31.8	p = 0.253	0 (n = 40)	108.8 ± 32.0	p = 0.779	p = 0.396
**(mg/dl)**	16 (n = 25)	111.4 ± 34.4		16 (n = 19)	110.7 ± 37.6		
							
**VLDL **	0 (n = 38)	21.9 ± 13.0	p = 0.026	0 (n = 37)	27.9 ± 17.9	p = 0.01	p = 0.652
**(mg/dl)**	16 (n = 24)	17.5 ± 9.8		16 (n = 20)	26.1 ± 16.4		
							
**Triglycerides **	0 (n = 39)	109.2 ± 63.7	p = 0.061	0 (n = 40)	134.7 ± 86.8	p = 0.009	p = 0.502
**(mg/dl)**	16 (n = 25)	91.8 ± 52.7		16 (n = 20)	130.4 ± 81.6		
							
**CRP **	0 (n = 38)	5.7 ± 4.8	p = 0.200	0 (n = 39)	7.1 ± 9.4	p = 0.018	p = 0.45
**(mg/L)**	16 (n = 25)	3.6 ± 2.9		16 (n = 20)	4.3 ± 4.7		
							
**ULP **	0 (n = 44)	6.1 ± 2.2	p = 0.684	0 (n = 44)	5.5 ± 1.7	p = 0.538	p = 0.973
**(micromol/g creatinine)**	16 (n = 27)	6.2 ± 2.2		16 (n = 20)	5.3 ± 2.4		

Δ = Change from baseline to 16 weeks.

^† ^= Secondary outcomes.

**Table 5:** Lipid and Inflammatory Measures at Week 0 and Week 40

Measurement^†^	Week	MD Group Mean ± SD	Within Group Δ p-value	Week	FB Group Mean ± SD	Within Group Δ p-value	Between Group Δ p-value
**Total**	0 (n = 39)	191.2 ± 39.5	p = 0.154	0 (n = 40)	187.5 ± 43.0	p = 0.003	p = 0.632
**Cholesterol (mg/dl)**	40 (n = 23)	182 ± 32.5		40 (n = 20)	183.0 ± 45		
							
**HDL **	0 (n = 39)	52.0 ± 16.0	p = 0.509	0 (n = 40)	51.8 ± 18.0	p = 0.029	p = 0.86
**(mg/dl)**	40 (n = 23)	53.2 ± 12.2		40 (n = 20)	49.6 ± 11.8		
							
**LDL **	0 (n = 39)	117.3 ± 31.8	p = 0.088	0 (n = 40)	108.8 ± 32.0	p = 0.099	p = 0.811
**(mg/dl)**	40 (n = 23)	107.1 ± 28.5		40 (n = 20)	104.1 ± 31.1		
							
**VLDL**	0 (n = 38)	21.9 ± 13.0	p = 0.614	0 (n = 37)	27.9 ± 17.9	p = 0.38	p = 0.648
**(mg/dl)**	40 (n = 23)	21.7 ± 14.0		40 (n = 20)	29.3 ± 16.4		
							
**Triglycerides **	0 (n = 39)	109.2 ± 63.7	p = 0.516	0 (n = 40)	134.7 ± 86.8	p = 0.391	p = 0.579
**(mg/dl)**	40 (n = 23)	107.7 ± 70.1		40 (n = 20)	137.4 ± 101.2		
							
**CRP**	0 (n = 38)	5.7 ± 4.8	p < 0.0001	0 (n = 39)	7.1 ± 9.4	p = 0.008	p = 0.886
**(mg/L)**	40 (n = 23)	2.6 ± 2.2		40 (n = 20)	3.5 ± 3.6		
							
**ULP **	0 (n = 44)	6.1 ± 2.2	p = 0.006	0 (n = 44)	5.5 ± 1.7	p = 0.837	p = 0.038
**(micromol/g creatinine)**	40 (n = 23)	4.9 ± 2.4		40 (n = 20)	5.4 ± 2.5		

Δ = Change from baseline to 40 weeks.

^† ^= Secondary outcomes.

### Weight

After the 16-week active weight loss phase, weight loss among completers averaged 12.3% (13.5 ± 5.9 kg) on the MD versus 6.7% (6.5 ± 6.8 kg) on the FB (p = 0.001). Twenty-six of 28 (92.9%) MD participants, lost ≥ 5% of their initial body weight at 16 weeks, versus 11 of 20 (55.0%) FB participants (χ^2 ^= 9.47, df = 1, p = 0.002). 21 of 28 (75%) MD participants lost ≥ 10%, versus 5 of 20 (25%) FB participants (χ^2 ^= 11.75, df = 1, p = 0.001). Over the16 weeks of active weight loss, BMI reduced from 38.5 to 33.8 kg/m^2^, an average decrease of 12.3% for the MD, and from 37.8 to 34.7 kg/m^2^, an average decrease of 6.7% for FB participants, representing a significant between group difference (Mann-Whitney U = 125, Z = -3.24, p = 0.001) (Table 2).

At week 40, after 24 weeks of weight maintenance, the MD group regained 4.8 ± 5.8 kg (Z = -3.565, p < 0.0001) of initial weight loss compared to the FB group that regained 0.8 ± 4.8 kg (Z = -0.728, p = 0.467); this was a significant between group difference (Mann Whitney U = 153, Z = -2.216, p = 0.027). However, both groups maintained significant weight loss (from baseline to week 40) with a mean net loss of 8.9 ± 8.9 kg (-7.8%; Z = -3.76, p < 0.0001) in the MD group and 5.7 ± 8.6 kg (-5.9%; Z = -2.86, p = 0.004) in the FB group. Significantly more participants in the MD group, 16 of 26 (61.5%), maintained ≥ 5% weight loss compared to only 6 of 20 (30%) in the FB group (χ^2 ^= 4.506, df = 1, p = 0.034). 10 of 26 (38.5%) MD participants maintained a ≥ 10% weight loss, versus 4 of 20 (20%) FB participants (χ^2 ^= 1.82, df = 1, p = 0.117). At week 40, BMI in the MD group remained reduced from baseline by 7.8% versus 5.9% in the FB group (Mann-Whitney U = 195, Z-1.44, p = 0.15) (Table 2).

### Body Fat Percentage and Lean Muscle Mass

During the 16-week weight loss phase, body fat % among the MD group decreased by a mean of 5.6%, representing a 13.6% reduction from baseline (Z = -454, p < 0.0001), whereas the FB group experienced a nonsignficant average decrease of 1.5%, representing a 2.7% reduction from baseline (Z = -1.107, p = 0.27). The between group difference for body fat % was statistically significant (Mann-Whitney U = 102.5, Z = -3.605, p < 0.0001). Lean Muscle Mass as a percent of total weight was significantly increased from baseline to Week 16 in the MD group (from 54.1% to 59.3%; Z = -427, p < 0.0001), whereas the FB group did not experience any significant change (Z = -0.97, p = 0.332). This difference was significant between groups (p < 0.0001) (Table 1).

At week 40, a mean body fat % decrease among the MD group was 2.9%, whereas the FB group decreased by 1.8% (p < 0.0001 and p = 0.10, respectively), representing a marginally significant between group difference (Mann Whitney U = 179, Z = -1.784, p = 0.07). Again, lean muscle mass as a percent of total weight was significantly increased by 4.5% from baseline to week 40 in the MD group (Z = -2.74, p = 0.006), whereas the FB group did not experience any significant change (Z = -1.38, p = 0.17). This was not a significant between group difference (p = 0.126) (Table 2).

### Waist Circumference and Visceral Fat Rating

During the 16-week weight loss phase, WC decreased by a mean of 13.0 cm (11.2%) in the MD group and 7.8 cm (6.8%) in the FB group (p = 0.003 and p < 0.0001, respectively). Visceral fat rating (VFR) was significantly reduced in the MD group, from a mean of 13.8 ± 3.8 at baseline to 10.6 ± 3.5 at 16 weeks, an average 25.4% reduction (Z = 4.315, p < 0.0001), while the FB group experienced an average marginal decrease of 3.7% (Z = 1.743, p = 0.081). This difference between group was significant (Mann Whitney U = 79, Z = 3.948, p < 0.0001) (Table 1).

At week 40, WC in the MD group decreased by a mean of 9.7 cm (8.4%) [p < 0.0001], compared to 3.8 cm (3.3%) [p = 0.12] of the FB group, representing a significant difference between group (Mann Whitney U = 125, Z = -2.23, p = 0.03). Both groups retained a significant decrease in VFR from baseline. VFR in the MD group decreased 14.7% (Z = 3.53, p < 0.0001), whereas the FB group decreased by 9.2% (Z = -2.064, p = 0.039) for a marginally significant between group difference (Mann Whitney U = 171.5, Z = -1.637, p = 0.10) (Table 2).

### Blood Pressure and Pulse

After the 16-week weight loss phase, both groups experienced statistically significant declines in both systolic and diastolic blood pressure. The MD group lowered systolic blood pressure by a mean of 10.9 mmHg (8.5%) versus 9.2 mmHg (7.1%) for the FB group (p < 0.0001 and p = 0.003, respectively). For diastolic blood pressure, the MD group experienced a mean 6.5 mmHg (7.6%) decline, versus a 5.2 mmHg (5.7%) decline for the FB group (p = 0.001 and p = 0.016, respectively). Both groups had significant decreases in pulse; a 10.7% reduction in the MD group (Z = -3.427, p = 0.001) and a 5.7% reduction in the FB group (Z = -2.538, p = 0.011) (Table 2).

At week 40, the MD group reduced systolic blood pressure by 6.0 mmHg (4.5%) and the FB group by 8.3 mmHg (6.5%) (p = 0.01 and p = 0.02, respectively). Diastolic blood pressure was decreased by 5.5 mmHg (6.2%) for the MD group, compared to 0.9 mmHg (0.45%) in the FB group (p = 0.01 and p = 0.62, respectively) for a statistically significant between group difference (Mann Whitney U = 173, Z = -1.93, p = 0.05). Both groups retained significant decreases in pulse at week 40. The MD group remained reduced by 7.9% (Z = -2.858, p = 0.004); the FB group by 4.5% (Z = -2.24, p = 0.025). There was no significant difference between the groups (Table 3).

### Satiety

There were no significant between group differences for either question assessing satiety at week 16. There was no difference in post-meal fullness (MD = 6.7 ± 2.0 vs. FB = 5.7 ± 2.1, p = 0.203) or general fullness (MD = 5.8 ± 1. vs. FB = 5.2 ± 2.2, p = 0.405).

### CRP

During the 16-week weight loss phase, CRP decreased in the MD group by a mean of 1.9 mg/dL (7.8%), compared to a 3.3 mg/dL (32.1%) decrease in the FB group (p = 0.20 and p = 0.018, respectively), however, there was no statistically significant between group differences observed (Table 4).

At week 40, CRP levels in the MD group decreased by a mean of 3.1 (40.7%), whereas the FB group decreased by a mean of 4.1 (30.1%) (p < 0.0001 and p = 0.01, respectively). There were no significant differences between groups (Table 5).

Regression was used to further examine the relationship between CRP levels at 40 weeks and predictor variables and confounders. A significant interaction between baseline CRP levels, intervention group and time was found (p = 0.04), even when controlling for significant confounders (i.e., birth control use and changes in cholesterol). A dichotomous variable was used to characterize baseline CRP levels (low levels defined as ≤ 3.0 mg/dL; high was defined as > 3.0 mg/dL). Both FB and MD groups with low baseline CRP levels experienced no significant changes over time. The FB group with high baseline CRP levels experienced marginally significant decreases over time (β = -5.83, p = 0.06); however the MD group with high baseline CRP levels was the only sub-group to experience significant decreases over the 40 weeks (β = -5.03, p < 0.0001).

### Urine Lipid Peroxides (ULP)

At 16 weeks, no significant within or between group differences in ULP were seen. At week 40, ULP levels in the MD group decreased a mean of 1.3 micromol/g creatinine (17.5%), compared to 0.2 micromol/g creatinine (5.4%) among the FB group (p = 0.01 and p = 0.84, respectively), which represented a significant difference between groups (Mann Whitney U = 145, Z = -2.07, p = 0.04) (Table 4).

Regression was also used to further examine the relationship between ULPs at 40 weeks with predictor variables and confounders. A significant interaction between intervention group and time was found (p = 0.05), even when controlling for significant confounders (i.e., age, gender, positive report of arthritis, birth control use and changes in cholesterol). The FB group did not experience any significant changes over the 40 weeks (β = 0.09, p = 0.84), whereas there was a significant mean decrease over time in the MD group (β = -1.26, p = 0.005) (Table 5).

### Cholesterol

At 16 weeks, both MD and FB groups nonsignificantly lowered total cholesterol levels by a mean of 0.6 ± 15% and 2.6 ± 16%, respectively. Similarly, neither group experienced significant changes in low density lipoprotein (LDL) or high density lipoprotein (HDL) cholesterol levels at 16 weeks. However, very low density lipoprotein (VLDL) levels were significantly decreased from baseline, in both the MD (-8.8%; Z = -2.23, p = 0.03) and FB groups (-15.3%; Z = -2.55, p = 0.01) (Table 4).

At week 40, only the FB group retained significant reductions in total cholesterol (-3.6%; Z = -2.99, p = 0.003). HDL cholesterol remained significantly increased in the FB group (up 6.1%, p = 0.03). Both groups retained marginally significant reductions in LDL cholesterol from baseline. MD group remained reduced by 4.4% (Z = -1.71, p = 0.09) and the FB group by 5.4% (Z = 1.65, p = 0.10). No significant changes in VLDL remained in either group. There were no significant between group differences for total cholesterol, HDL, LDL, or VLDL at week 40 (Table 5).

### Triglycerides

After the initial 16-week weight loss phase, both groups reduced fasting triglycerides; the MD group reduced triglycerides by 4.6% (Z = -1.87, p = 0.06) and the FB group by 15.3% (Z = 2.60, p = 0.01) (Table 4). At week 40, there were no significant differences from baseline in either group. There were no significant between group differences at either time point (Table 5).

## Discussion

Increasingly, meal replacement diet plans have been demonstrated to provide safe, effective, sustainable weight loss, and have also been shown to yield significant improvements in health outcomes [8,9,13-15]. Nutrient rich, portion-controlled meal replacements are a strategic tool that may assist dieters as they navigate the obesigenic environment by providing a convenient alternative to over-sized, high fat, empty calorie choices [23]. For these reasons, this study sought to evaluate the impact of a portion-controlled meal replacement diet plan on body weight and body composition compared to an isocaloric, food-based diet plan for a 16-week period of weight loss and 24-week period of weight maintenance.

Following a low-energy diet consisting of five, 90-110 kcal meal replacements daily and one self-prepared meal (MD group) led to twice the weight loss at the end of 16-weeks compared to a food group prescribed the same number of calories based on food selection guidelines of the USDA Food Guide Pyramid. Clinically significant weight loss, as defined by the Institute of Medicine (IOM), is a loss of at least 5% of starting body weight in one year [26,27]; 93% of participants following the MD diet compared to only 55% of the FB group achieved this in a 10-month time period. Moreover, a robust mean weight loss of 12.3% was observed among the MD group after 16 weeks, a magnitude many drugs currently used for obesity pharmacotherapy do not achieve [28].

While there was not a significant difference between groups in absolute weight loss at 40 weeks, and weight regain during maintenance was greater among the MD group than it was for the FB group, clinically significant weight loss was maintained by considerably more of the MD group (61.5%), compared to the FB group (30%). Overall, the MD group maintained a mean net loss from baseline approximately 2% greater than the FB group (-7.8% (-8.9 ± 8.9 kg) and -5.9% (-5.7 ± 8.5 kg) respectively).

Significant improvements in body composition were also observed in the MD group compared to the FB group after 16 weeks of weight loss. MD participants lost five times more body fat and seven times more visceral fat, while maintaining more than twice the amount of lean muscle mass. Maintenance of lean muscle mass during weight loss on a hypocaloric diet is an important difference between the meal replacement diet plan under study and other weight loss plans [29]. Sustaining lean muscle mass is a crucial mechanism for maintaining weight loss, as muscle provides a higher contribution to resting metabolic rate (RMR) than does fat [30-32]. A likely explanation for the favorable body composition changes observed in the MD group is the macronutrient composition (low fat, low carbohydrate, higher protein) of the meal replacements, which is difficult to achieve without significant planning when dieters self-prepare meals.

After 16 weeks of weight loss and another 24 weeks of weight maintenance, both groups experienced improvements in biochemical outcomes and other clinical indicators of health, like blood pressure and pulse. At 40 weeks, significant differences in the magnitude of improvement in biomarkers of cardiovascular health emerged in the MD group compared to the FB group. Significant improvements in diastolic blood pressure, waist circumference, and oxidative stress were found only in the MD group. Concentrations of CRP were also significantly decreased from baseline in the MD group, especially among those with high baseline CRP. Mechanistically, decreases in total fat, visceral fat, and waist circumference may be responsible for the decreases seen in inflammation and oxidative stress, as abdominal fat has been shown to produce inflammatory molecules that underlie metabolic syndrome and cardiovascular disease [33]. This is highlighted by recent research which found central obesity to be an independent predictor of coronary heart disease and cardiovascular disease deaths [34], and waist circumference alone as a very good predictor of health risk and mortality in overweight and obese individuals [35,36].

A possible factor contributing to the greater overall effectiveness for initial weight loss on the meal replacement diet plan studied is ease of use for the end-user, leading to enhanced compliance with the diet plan. Better adherence to the diet using meal replacements has been shown over both the short-term and long-term [8,9] as well as among subgroups of individuals, such as those with type 2 diabetes, who are often challenging in terms of compliance and achievement of weight loss [8]. While not statistically significant, this was demonstrated by the greater number of the MD group completing the both the 16-week weight loss phase and 24-week weight maintenance phase. Lack of significant between group differences in completion rate may have been muted by the high and equal dietary support given to both groups, as well as the relatively small study sample size.

While the results of this study are compelling, limitations do exist. The overall drop-out rate of 43.2% for the MD group after ten months, is a limitation of the study, though it is consistent with the rates found for four of the most common diet plans studied for only a two-month period: Atkins (47%), Ornish (50%), Weight Watchers (35%), and Zone (35%) [37]. Dietary fatigue, as a result of using the same or similar meal replacements, could be a factor in the drop-out rate of the MD group, however, this drop-out rate was similar to a recent study using MD meal replacements over a comparable time frame [8]. The higher completion rate in the MD group, although not significant, may have been affected by meal replacements being provided during the study, whereas participants in the FB group were responsible for providing their own food. In addition to the provision of meal replacements during the study, the dietitian/participant relationship could have affected individual results, compliance, and completion of the study. As stated previously, it was impossible to blind subjects to the intervention and it is possible being randomized to an undesired group, also affected individual results, compliance, and completion of the study. Overall, the drop-out rate was higher than expected given the close follow-up with dietitians. Finally, while this study reports significant findings for many secondary outcomes (as shown in Tables 2, 3, 4, 5), it was only powered to detect the primary outcomes of body weight and body composition.

## Conclusions

In conclusion, we found that a meal replacement diet plan of a fixed macronutrient composition yielded clinically significant weight loss for 93% of obese participants. This is roughly twice as much as the rate demonstrated in controlled clinical trials of currently approved pharmacologic agents for obesity treatment [28]. Also, the intervention with meal replacements yielded changes in body composition that favorably impacted many cardiovascular health outcomes. Our data suggest that the meal replacement diet plan evaluated is an effective strategy for producing robust initial weight loss, and for achieving improvements in a number of health parameters during weight maintenance, including inflammation and oxidative stress, two key factors recently understood to underlie our most common chronic diseases.

## Competing interests

LMD, CC, JK, TH, LF, and JR are salaried employees of the sponsoring institution, Medifast, Inc.

WSA holds stock in the sponsoring institution, Medifast, Inc.

## Authors' contributions

LMD conceived of the study, and participated in its design, coordination, acquisition of data, and drafted the manuscript. CC participated in its conception, design, coordination, acquisition of data, and helped to draft and revise the manuscript. JK participated in the acquisition of data and manuscript revision. TH, LF, and JR participated in its coordination and data acquisition. WSA participated in the conception and design and was the medical monitor providing medical oversight of study participants. AHM performed the statistical analyses, interpreted the data, and helped draft the manuscript. All authors read and approved the final manuscript.
